# Understanding Postprandial Inflammation and Its Relationship to Lifestyle Behaviour and Metabolic Diseases

**DOI:** 10.1155/2012/947417

**Published:** 2011-09-25

**Authors:** Boudewijn Klop, Spencer D. Proctor, John C. Mamo, Kathleen M. Botham, Manuel Castro Cabezas

**Affiliations:** ^1^Department of Internal Medicine, Center for Diabetes and Vascular Medicine, Sint Franciscus Gasthuis, 3004 BA Rotterdam, The Netherlands; ^2^Metabolic and Cardiovascular Diseases Lab, Molecular Cell Biology of Lipids Group, Alberta Diabetes and Mazankowski Alberta Heart Institutes, University of Alberta, Edmonton, AB, Canada T6g2R3; ^3^Faculty of Health Sciences, Curtin University, Perth and Sydney, WA 6102, Australia; ^4^Department Of Veterinary Basic Sciences, The Royal Veterinary College, London NW1 0TU, UK

## Abstract

Postprandial hyperlipidemia with accumulation of remnant lipoproteins is a common metabolic disturbance associated with atherosclerosis and vascular dysfunction, particularly during chronic disease states such as obesity, the metabolic syndrome and, diabetes. Remnant lipoproteins become attached to the vascular wall, where they can penetrate intact endothelium causing foam cell formation. Postprandial remnant lipoproteins can activate circulating leukocytes, upregulate the expression of endothelial adhesion molecules, facilitate adhesion and migration of inflammatory cells into the subendothelial space, and activate the complement system. Since humans are postprandial most of the day, the continuous generation of remnants after each meal may be one of the triggers for the development of atherosclerosis. Modulation of postprandial lipemia by lifestyle changes and pharmacological interventions could result in a further decrease of cardiovascular mortality and morbidity. This paper will provide an update on current concepts concerning the relationship between postprandial lipemia, inflammation, vascular function, and therapeutic options.

## 1. Introduction

Atherosclerosis is the primary cause of death in the world [1]. Classical risk factors such as smoking, hypertension, fasting hyperlipidemia, insulin resistance, increased body fat mass, and unfavourable body fat distribution are strongly interrelated and can often be found in one and the same subject. Subjects with fasting hypertriglyceridemia usually have elevated postprandial lipids due to the close correlation of fasting and postprandial triglycerides (TG) [2]. Postprandial lipemia has gained interest because of recent reports showing that nonfasting TG independently predict the risk for atherosclerosis [3, 4] and are possibly even stronger predictors of cardiovascular disease (CVD) than fasting TG [3, 5]. 

Atherosclerosis is considered a low-grade chronic inflammatory disease [6], and both the postprandial phase and chronic disease states such as the metabolic syndrome are associated with increased inflammation. This paper outlines recent developments in the understanding of postprandial inflammation and its relationship with vascular function, metabolic diseases, and lifestyle behaviour.

## 2. Metabolism of Postprandial Lipemia

Dietary fat is absorbed in the intestine and secreted into lymph by enterocytes in TG-rich chylomicrons. Once in the circulation, chylomicrons rapidly undergo hydrolysis to produce cholesterol-dense lipoprotein remnants which are taken up by the liver [7, 8]. After a fatty meal, exogenous fatty acids are delivered to the liver by chylomicron remnants and may then be reassembled and returned to the blood in very low-density lipoproteins (VLDL) [9]. The hypertriglyceridemia observed postprandially is due to raised concentrations of chylomicrons, VLDL, and their respective remnants, collectively known as triglyceride-rich lipoproteins (TRLs).

People in the Western world are nonfasting for most of the day, consequently leading to a continuous challenge of the endothelium by atherogenic lipoprotein remnants [10, 11]. The exogenous chylomicrons and endogenously produced VLDL share the same metabolic pathway, for example, endothelium-bound lipoprotein lipase (LPL), which hydrolyzes TG into glycerol and fatty acids. In the postprandial phase, due to limited LPL availability, competition at the level of this enzyme will occur resulting in accumulation of TRLs. This competition is most likely when fasting hypertriglyceridemia is present. The increased levels of free fatty acids (FFAs) as a result of a hypercaloric diet are regarded as one of the key etiologic components of the metabolic syndrome, type 2 diabetes (T2DM), and obesity [12, 13].

## 3. Residual Risk of Cardiovascular Disease after LDL Cholesterol Lowering

Based on results from large clinical trials, lipid management for reducing the risk for CVD has been typically focused on reducing LDL-C by statin therapy [14–18]. Despite aggressive LDL-C lowering by statin therapy, approximately two-thirds of all CVD events remain. These “residual” events appear to be independent of the LDL-C and in recent years have gained momentum as a concept of “residual risk” of CVD. Interestingly, statins do reduce postprandial lipemia and also have an effect on complement, but they do not affect TG sufficiently to be of clinical relevance in hypertriglyceridemic conditions [2, 19–22]. Interestingly, this “residual risk” has been found to be greater for treated patients with diabetes or the metabolic syndrome than in untreated patients without these conditions [23, 24]. One could interpret these observations to infer that statin therapy, resulting in LDL-C lowering, does not necessarily bring the relative CVD risk in patients suffering from diabetes and metabolic syndrome to the level of nondiabetics and patients without metabolic syndrome. Consequently, the current model we propose is that the “residual risk” hypothesis of atherosclerosis is not just dependent on circulating concentrations of LDL-C but is equally dependent on remnant lipoprotein concentrations and perturbations in the arterial vessel wall that influence the rate of arterial lipoprotein retention. The potential impact of the “residual risk” hypothesis is perhaps most appreciated during conditions of increased atherosclerotic risk. For example, subjects with insulin resistance, T1DM, or T2DM showed raised plasma concentrations of fasting remnant lipoproteins and an ensuing impairment in postprandial lipoprotein metabolism [25, 26]. The remnant lipoproteins are able to penetrate arterial tissue and become entrapped within the subendothelial space [27]. It has also been demonstrated that remnant lipoproteins can induce macrophage lipid loading, which is a hallmark feature of early atherogenesis [28–30]. Moreover, raised fasting concentrations of apoB48, the specific protein of chylomicrons and their remnants [31], have been shown to be elevated in patients with obesity, insulin-resistance, and T2DM [32–35]. Numerous studies have shown that fasting levels of remnant lipoproteins can predict impaired metabolism of chylomicrons, particularly in those at risk of CVD [36–38]. Under experimental conditions, some studies have suggested that the small LDL particles may have a higher rate of delivery but in turn efflux more readily from arterial tissue compared to remnant lipoproteins [39, 40]. However, despite many studies showing atherogenic effects of impaired postprandial lipemia, it remains uncertain whether lowering postprandial lipemia would reduce CVD risk or if TG are merely a marker of other metabolic abnormalities [41]. But it is certain that postprandial lipemia, obesity, insulin resistance, inflammation, and vascular function and atherogenesis are closely related with each other.

## 4. Postprandial Lipemia and Vascular Integrity

**Figure 1:**
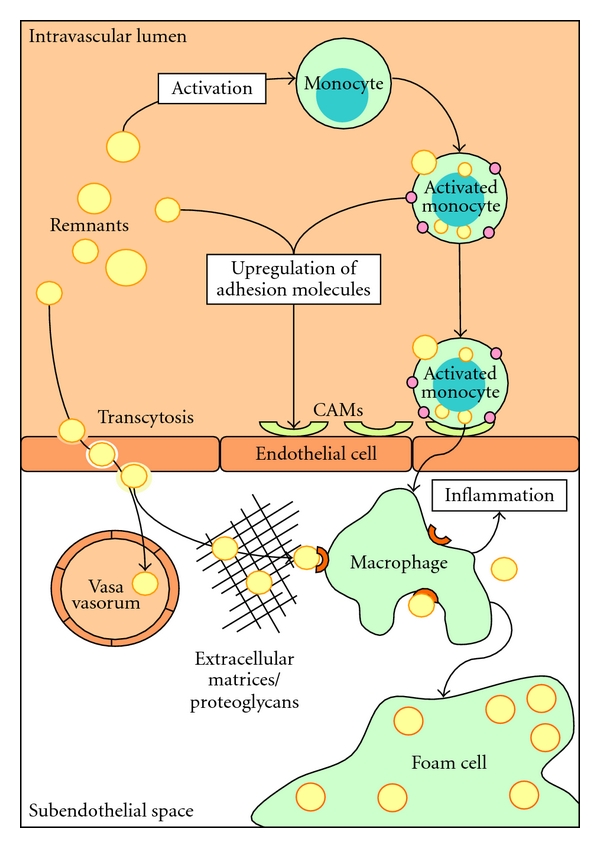
Concept of the initiation of atherosclerosis by remnant lipoproteins: remnants enter the subendothelial space via nonspecific transcytotic processes. This is often a nonpathologic process, because the remnants leave the subendothelial space again via the vasa vasorum. However, retention of remnants may occur in the presence of proteoglycans and excess extracellular matrices. Remnants can be easily taken up by macrophages, in contrast to LDL, which need to become modified first. Circulating remnants themselves also contribute to the presence of subendothelial macrophages. Monocytes can bind and take up remnants, which stimulates the monocytes to become activated. Subsequently, activated monocytes express adhesion molecules on the outer membrane and stimulate the expression of endothelial cellular adhesion molecules (CAMs), which allows monocytes to home on the endothelium and migrate into the subendothelial space. Finally, the macrophages change into highly atherogenic foam cells when lipid uptake exceeds lipid efflux.

Coronary arteries are characterized by tightly apposed endothelial cells with significant expression of tight junction proteins [42]. In healthy vessels, the coronary endothelium prevents the diffusion of large or hydrophilic molecules, thereby minimising extravasation of systemically derived potentially inflammatory agents and macromolecules. However, some lipoprotein transport including remnants of TRLs occurs across intact and functional endothelium via nonspecific transcytotic processes [40]. This phenomenon is predominantly non-pathogenic, because the lipoproteins are internalized via receptor processes or passage through the basal laminae and exit via the vasa vasorum. However, experimental evidence suggests that prolonged retention of TRLs as a consequence of binding to extracellular matrices and proteoglycans can stimulate chemotaxis and activation of circulating inflammatory cells (Figure 1) [43]. 

Dietary-lipotoxicity is a term commonly used to broadly describe processes leading to end-organ damage following excess exposure to particular lipids [44]. First identified in the context of fat-induced insulin resistance, the process has since been implicated in a range of chronic diseases and inflammatory disorders. Endothelial cells may be particularly susceptible to the effects of dietary lipids associated with TRLs because of the significant level of lipoprotein processing that occurs via interaction with hydrolytic lipases and, thereafter, the constant exposure to plasma fatty acids and cholesterol. Animal feeding studies have shown that saturated fatty acids and cholesterol-enriched diets increase protein oxidation and lipid peroxidation and significantly alter cell membrane phospholipids and lipid raft composition, key regulators of inflammation [45, 46]. Excess cholesterol can cause mitochondrial dysfunction and trigger apoptotic pathways [47]. Other mechanisms for dietary fat-induced alterations in cell function include stimulation of NADPH-oxidase-derived reactive oxygen species or modulation of mTOR (mammalian target of rapamycin), a key signal transduction protein that regulates vascular endothelial fenestration [48]. Many other factors have been suggested to participate in the generation of oxidative stress. Paraoxonase 1 (PON-1), a potent antioxidant closely associated with HDL-C, seems to be a key player [49]. During postprandial lipemia, HDL-C tends to decrease, impairing the reverse cholesterol transport and reducing the anti-inflammatory properties of HDL-C [50], again providing an extra atherogenic mechanism for postprandial lipemia. 

Commensurate with dietary fat modulation, the particle phenotype of lipoproteins determines the susceptibility to subendothelial retention. The heparin sulphate proteoglycans that bind apo B lipoproteins may have greater affinity for TRL remnants because of cooperative apolipoprotein binding domains principally between apo B and apo E and exacerbates as a consequence of diabetes [51]. Moreover, apo E facilitates unabated uptake of remnant lipoproteins by macrophages via alternate pathways without the requisite of lipoprotein modification such as oxidation [52, 53]. 

Nitric oxide (NO) is also one of the key players of endothelium-derived factors, which influences vasomotion, permeability, proliferation, and vascular smooth cell migration [54]. NO-mediated endothelial-dependent vascular relaxation has been shown to be impaired by remnant lipoproteins in studies with isolated vessel segments from rats and pigs *in vitro* [55]. In human subjects with the metabolic syndrome, but also in healthy subjects, elevated fasting and postprandial TG have been related to increased carotid intima-media thickness (IMT) [56] and reductions in NO-dependent postischemic flow-mediated dilation (FMD) of the brachial artery [57, 58]. This reduction of FMD correlated with TG and FFA concentrations and was reversible when TG concentrations decreased at the end of the oral fat loading test [57]. Furthermore, postprandial TRLs have been shown to induce the expression of leukocyte adhesion molecules on the endothelium, facilitating recruitment of inflammatory cells [59] and remnant lipoproteins have been found to activate endothelial cells by upregulating COX-2 expression and activating intracellular signaling pathways controlled by nuclear factor-kappaB and mitogen-activated protein kinases [60].

## 5. Triglyceride-Rich Lipoproteins and Inflammation

Many inflammatory markers, such as C-reactive protein (CRP), leukocyte count, and complement component 3 (C3), have been associated with CVD [61–66]. Furthermore, several studies with animal models showed reduced plaque formation [67, 68] and prevention of endothelial dysfunction [69], when adherence of leukocytes to the endothelium was prevented. These findings support the theory that atherogenesis, in part, starts with leukocyte-endothelium interaction and adherence. Obligatory for this adherence is a cytokine-controlled sequential upregulation of selectins and adhesion molecules on activated leukocytes and endothelial cells [70]. 

Van Oostrom et al. have shown that postprandially, when TG and glucose rise, neutrophil counts increase with concomitant production of pro-inflammatory cytokines and oxidative stress; and that these changes may contribute to endothelial dysfunction [71, 72]. Furthermore, TG and glucose are able to induce leukocyte activation, as has been shown *in vitro *[73, 74] and *ex vivo* in hypertriglyceridemic patients [75]. In healthy volunteers and in patients with premature CVD, postprandial lipemia has been associated with the upregulation of leukocyte activation markers [22, 76]. Fasting leukocytes of patients with CVD have an increased lipid content when compared to controls, and it has been suggested that this is due to uptake of chylomicrons [77]. Furthermore, uptake of remnant lipoproteins by primary human monocytes has been demonstrated in experiments *in vitro* [74]. Leukocytes are also able to take up retinyl esters, as markers of intestinally derived TRLs [78]. Recently, we have shown that apo B binds to neutrophils and monocytes and that postprandial leukocytes transport dietary fatty acids [79]. This opens the possibility that direct activation of leukocytes may occur in the blood by interaction with chylomicrons and their remnants (Figure 1). 

Another inflammatory pathway related to CVD and lipid metabolism is the complement system. The C3/acylation stimulating protein- (C3/ASP-) system has been recognized as a regulator of adipose tissue fatty acid metabolism [80]. ASP is identical to the desarginated form of the C3 split-product C3a (C3a-desArg), which is immunologically inactive. The C3/ASP pathway stimulates re-esterification of FFA into TG in adipocytes, reduces adipocyte FFA production by inhibiting hormone sensitive lipase and stimulates glucose uptake by adipocytes, fibroblasts, and muscle cells [80]. C3 is a strong predictor of myocardial infarction [64], and it has been positively associated to obesity, CVD, insulin resistance, the metabolic syndrome [81], fasting and postprandial TG, and hypertension [2, 64]. Complement components have been shown to colocalize with CRP in atherosclerotic plaques [82] and complement activation also plays a role in the induction of tissue damage after myocardial infarction [83]. Moreover, chylomicrons are the strongest *in vitro* and *in vivo* stimulators of adipocyte C3 production via activation of the alternative complement cascade [66, 84]. A postprandial C3 increment after a fat meal has been shown in healthy subjects, patients with CVD, and patients with familial combined hyperlipidemia [2, 63, 66]. Moreover, this postprandial increment has been related to TG and FFA metabolism [85].

## 6. Metabolic Syndrome and Insulin Resistance in Relation to Atherosclerosis and Postprandial Lipemia

Insulin resistance has also been shown to be associated with impaired vasodilatation, increased oxidative stress and increased concentrations of FFAs, vasoconstrictors, cell adhesion molecules, cytokines, and several other mediators of low-grade inflammation and thrombogenesis [86]. Insulin resistance increases the risk for CVD severalfold compared to the normal population; however, the underlying mechanisms are not completely defined [87]. Insulin resistance often clusters with elevated blood pressure, obesity, central obesity, elevated TG, and low HDL-C. However, whether hyperinsulinemia itself is indeed an independent predictor of CVD has often been debated [87]. A recent meta-analysis by Ruige et al. showed a weak positive association between high insulin levels and CVD events [88]. Another meta-analysis involving 87 studies, which included 951,083 patients based on the definitions of metabolic syndrome by the 2001 National Cholesterol Education Program (NCEP) and 2004 revised National Cholesterol Education Program (rNCEP) demonstrated a 2-fold increase in cardiovascular outcomes and a 1.5-fold increase in all-cause mortality in subjects with the metabolic syndrome [89]. According to the NHANES III data, subjects with metabolic syndrome but without diabetes had a significantly increased prevalence of CVD [90]. However, recently, it was shown that the metabolic syndrome could not improve prediction of intima media thickness progression compared to the sum of its risk components [91]. It is evident that postprandial lipemia is prevalent during conditions of obesity and insulin resistance and may contribute to increased progression of CVD. However, a significant clinical dilemma still exists in diagnosing the early phases of the metabolic syndrome (i.e., prediabetes) and how this impacts on relative risk of CVD. In part, this has been impaired by the continued emphasis on LDL-C, which is often normal during early T2DM, leading to undetected yet insidious progression of CVD [92, 93]. Indeed, it is interesting to note that the recent revision by the IDF (International Diabetes Federation) has defined the metabolic syndrome independent of LDL-C concentrations [94]. In general, in clinical practice, the positive effects of LDL-lowering therapy on atherosclerosis and CVD are nowadays undisputable. While these efforts are well documented, much less is known about the clinical benefits of treating postprandial lipemia, despite increasing evidence supporting a causal role between remnant lipoproteins and the development of CVD [95]. Clinical studies have so far failed to provide a definitive association between impaired postprandial lipoprotein metabolism and the very early phases of insulin resistance and corresponding risk indices. Thus, animal models have to offer further characterization of the early stages of metabolic syndrome in order to understand the metabolic and postprandial profile of this condition. Despite a greater emphasis on the study of CVD risk in the metabolic syndrome, there remains a lack of well-characterized prediabetic models in order to investigate the role of postprandial lipoprotein metabolism in the development of atherosclerosis.

## 7. Diet, Lifestyle, Pharmacotherapy, and Postprandial Lipemia

**Figure 2:**
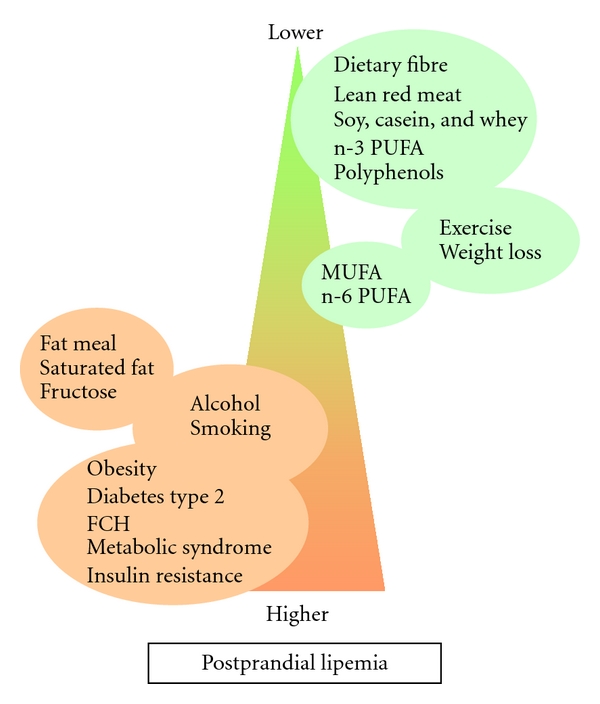
Influence of metabolic diseases and lifestyle factors on postprandial lipemia: factors listed in green circles reduce postprandial lipemia, whereas the factors in red have detrimental effects on postprandial lipemia.

Postprandial hyperlipidemia has many negative effects on vascular integrity, inflammation, and fatty acid metabolism but can be positively influenced by diet and lifestyle behaviour. Since postprandial lipemia is a physiological response to a fatty meal, it could be predicted that it would be influenced by the amount and type of fat in the diet, and there is strong evidence to support this [96, 97]. However, in recent years, it has become clear that other lifestyle factors, including dietary protein, fibers and micronutrients, alcohol consumption, exercise, and smoking also play a significant role in the regulation of postprandial lipemia [96]. Postprandial hyperlipidemia may be a link between lifestyle choices and the current alarming rise in the incidence of obesity, insulin resistance, T2DM, and CVD [98]. A summary of the positive and negative effects of lifestyle factors and metabolic diseases on postprandial lipemia is shown in Figure 2.

Postprandial lipemia is evident after a fat meal containing >30 g fat and the rise in plasma TG is dose dependent up to about 80 g [96, 99]. Since the average content of Western style meals is 20–40 g fat and 3-4 meals/day are typically consumed, it can be concluded that postprandial lipemia is likely to be present for 18 h/day in the Western population [100]. A single fatty meal causes changes in TRL particle characteristics, such as their size, number, and apolipoprotein composition, which depend on the fatty acid composition of the food. The most pronounced lipemia judged by these criteria is caused by a meal containing saturated fatty acids (SFAs), which are found in high amounts in animal fat, followed by monounsaturated fatty acids (MUFA), the main fatty acids in olive oil, with polyunsaturated fatty acids (PUFA), which are found in vegetable (n-6 PUFA) and fish (n-3 PUFA) oils, causing the least pronounced effect [96]. n-3 PUFA have also been shown to cause a lower rise in postprandial lipemia compared to the other types of fat [96, 101, 102]. While acute studies provide useful information, changes in dietary habits need to be sustained in the long term for beneficial effects on health. Both chronic intake and dietary supplementation of n-3 PUFA for periods varying from 4 weeks to 6 months have been shown to decrease postprandial hyperlipidemia due to decreased production of TRLs [103–106]. The effects of fatty acids other than n-3 PUFA on postprandial lipemia are less well defined, but generally, MUFA or n-6 PUFA as compared to SFA have been found to be more beneficial [96, 107, 108]. 

In addition to fat, the type of dietary proteins and carbohydrates may also influence postprandial lipemia. Lean red meat, soy protein, casein, and whey protein have all been associated with a reduced postprandial lipemic response [96, 107, 109], as have indigestible carbohydrates (i.e., dietary fiber) in the form of oat bran, wheat fiber, wheat germ, or psyllium husk [96, 101, 110]. Digestible carbohydrates, on the other hand, appear to have little effect [111] except for fructose which may enhance the postprandial lipemic response if more than 50 g per day are consumed [112].

Besides macronutrients like fats, carbohydrate, and protein, the diet contains micronutrients including vitamins, carotenoids, plant sterols, and polyphenols found in fruit and vegetables and in beverages such as green tea and red wine, and these are believed to contribute to the protective effect against CVD [113]. Polyphenols in green tea and strawberries have been reported to reduce postprandial lipemia in hyperlipidemic individuals [114, 115]. In addition, micronutrients in olive oil have been found to reduce postprandial lipemia [116]; however, no evidence for an effect of plant sterols on postprandial lipemia was found in a study with patients on lipid lowering therapy [117]. 

In addition to changing to a healthier diet, weight loss and increased physical activity are effective lifestyle interventions which reduce postprandial lipemia [118–120]. Exercise before a fat meal, even if only low-to-moderate volume, has been found to decrease postprandial lipemia in many studies [120–125]. Moreover, moderate and high exercise bouts appear to be equally beneficial [126], and a recent meta-analysis of 16 studies concluded that exercise in short bouts is as effective as continuous exercise in lowering postprandial blood TG concentrations [127]. Low-volume exercise, however, has been reported to be ineffective in smokers [128], and Bloomer et al. have also reported recently that long-term high-volume exercise had no effect on postprandial lipemia in young, healthy individuals [129]. Combining increased physical activity with dietary changes such as increased n-3 PUFA intake has been found to have a synergistic effect in reducing postprandial lipemia in active individuals [96]. The reduction in postprandial lipemia caused by exercise is believed to be due to increased clearance of TRLs, which is at least partially mediated by an increase in LPL activity [96]. However, current evidence suggests that it is not accompanied by a decrease in the associated postprandial inflammation, as assessed by markers such as C reactive protein, IL-6, or adhesion molecules [121, 123, 124].

In addition to daily meals and exercise, alcohol and smoking also influence postprandial lipemia. A study by Sharrett et al., which included >600 subjects with or without CVD, demonstrated that smoking and alcohol consumption as well as the diet are good predictors of postprandial lipid levels [100]. Despite considerable evidence that low-to-moderate alcohol intake protects against CVD [130], both ethanol and red wine have been shown to cause a marked increase in postprandial lipemia when added to a test meal [96, 131]. Habitual smokers also have increased postprandial lipemia, and this is thought to be due to impaired clearance of chylomicrons and their remnants [132]. 

Besides lifestyle interventions, no large improvements have been made in treating postprandial lipemia with pharmacotherapy. Statins are highly effective in reducing LDL cholesterol, but they do not affect TG sufficiently to be of clinical relevance in hypertriglyceridemic conditions. However, rosuvastatin is able to reduce the postprandial proinflammatory and procoagulant changes in subjects with CVD [22]. In addition, a decrease in hepatic FFA flux has also been reported [133]. These independent effects from rosuvastatin may protect against CVD when hyperlipidemia is present. In contrast to statins, pharmacotherapy with fibrates is effective in lowering TG concentrations. Despite hypertriglyceridemia being common in the Western population, fibrates are used in only 3.6% of hypertriglyceridemic subjects [134]. Controversy remains in the effectiveness of fibrates on cardiovascular morbidity and mortality. Recently, a large meta-analysis of fibrates with 45,058 participants was performed [135]. This study showed a modest but significant relative risk reduction of 10% for major cardiovascular events and 13% reduction in coronary events, but mortality remained unaltered. While certain drugs are beneficial for improving insulin resistance and potentially postprandial lipemia, in specific groups of patients, the effects on chylomicron and remnant metabolism may be detrimental. For example, rosiglitazone increases postprandial accumulation of atherogenic remnants in HIV-infected patients with lipodystrophy [136]. Therefore, when evaluating the effects of specific pharmacotherapeutic interventions on postprandial lipemia, detailed information on all aspects of the potentially harmful situations will be needed. At this stage, there is no data available on comparative studies regarding lifestyle modification versus lipid lowering therapy on the modulation of postprandial lipemia.

## 8. Conclusions

A residual risk for CVD remains despite aggressive LDL-C lowering by statins, which can partly be explained by postprandial hyperlipidemia, which leads to several metabolic dysfunctions and dietary lipotoxicity via different several mechanisms. First of all, TRLs are able to penetrate the arterial wall leading to endothelial lipid deposits, attraction of monocytes within the subendothelial space, production of inflammatory markers, and oxidative stress. Secondly, obesity worsens insulin resistance which further increases postprandial lipemia, consequently resulting in a vicious circle. Lifestyle interventions like the type of diet, cessation of smoking, and weight loss are effective methods to reduce postprandial lipemia and its related dietary lipotoxicity. Statins also have a beneficial effect on postprandial lipemia. However, at this stage, there are no data available comparing the magnitude of lifestyle interventions with pharmacotherapy on chylomicron metabolism and reduction of CVD.
